# Plasmodium falciparum Resistance to a Lead Benzoxaborole Due to Blocked Compound Activation and Altered Ubiquitination or Sumoylation

**DOI:** 10.1128/mBio.02640-19

**Published:** 2020-01-28

**Authors:** Kirthana M. V. Sindhe, Wesley Wu, Jenny Legac, Yong-Kang Zhang, Eric E. Easom, Roland A. Cooper, Jacob J. Plattner, Yvonne R. Freund, Joseph L. DeRisi, Philip J. Rosenthal

**Affiliations:** a Department of Medicine, University of California, San Francisco, San Francisco, California, USA; b Department of Biochemistry and Biophysics, University of California, San Francisco, San Francisco, California, USA; c Chan Zuckerberg Biohub, San Francisco, California, USA; d Anacor Pharmaceuticals, Inc., Palo Alto, California, USA; e Dominican University of California, San Rafael, California, USA; National Institutes of Health

**Keywords:** malaria, *Plasmodium falciparum*, drug, benzoxaborole, resistance, PfPARE, ubiquitination, sumoylation, antimalarial agents, drug resistance evolution, drug resistance mechanisms

## Abstract

New antimalarial drugs are needed. The benzoxaborole AN13762 showed excellent activity against cultured Plasmodium falciparum, against fresh Ugandan P. falciparum isolates, and in murine malaria models. To gain mechanistic insights, we selected *in vitro* for P. falciparum isolates resistant to AN13762. In all of 11 independent selections with 100 to 200 nM AN13762, the 50% inhibitory concentration (IC_50_) increased from 18–118 nM to 180–890 nM, and whole-genome sequencing of resistant parasites demonstrated mutations in prodrug activation and resistance esterase (PfPARE). The introduction of PfPARE mutations led to a similar level of resistance, and recombinant PfPARE hydrolyzed AN13762 to the benzoxaborole AN10248, which has activity similar to that of AN13762 but for which selection of resistance was not readily achieved. Parasites further selected with micromolar concentrations of AN13762 developed higher-level resistance (IC_50_, 1.9 to 5.0 μM), and sequencing revealed additional mutations in any of 5 genes, 4 of which were associated with ubiquitination/sumoylation enzyme cascades; the introduction of one of these mutations, in SUMO-activating enzyme subunit 2, led to a similar level of resistance. The other gene mutated in highly resistant parasites encodes the P. falciparum cleavage and specificity factor homolog PfCPSF3, previously identified as the antimalarial target of another benzoxaborole. Parasites selected for resistance to AN13762 were cross-resistant with a close analog, AN13956, but not with standard antimalarials, AN10248, or other benzoxaboroles known to have different P. falciparum targets. Thus, AN13762 appears to have a novel mechanism of antimalarial action and multiple mechanisms of resistance, including loss of function of PfPARE preventing activation to AN10248, followed by alterations in ubiquitination/sumoylation pathways or PfCPSF3.

## Abstract (precis)

Benzoxaboroles are under study as potential new drugs to treat malaria. One benzoxaborole, AN13762, has potent activity and promising features, but its mechanisms of action and resistance are unknown. To gain insights into these mechanisms, we cultured malaria parasites with nonlethal concentrations of AN13762 and generated parasites with varied levels of resistance. Parasites with low-level resistance had mutations in PfPARE, which processes AN13762 into an active metabolite; PfPARE mutations prevented this processing. Parasites with high-level resistance had mutations in any of a number of enzymes, mostly those involved in stress responses. Parasites selected for AN13762 resistance were not resistant to other antimalarials, suggesting novel mechanisms of action and resistance for AN13762, a valuable feature for a new class of antimalarial drugs.

## INTRODUCTION

Malaria remains the most important parasitic disease in the world. In 2017, an estimated 219 million illnesses and 435,000 deaths were caused by malaria parasites, mostly Plasmodium falciparum (1). Although the malaria burden decreased considerably in the early 21st century, the decline appears to have stalled in recent years. Major challenges to malaria control include resistance to all major classes of antimalarials, including widely used artemisinin-based combination therapies (2). Therefore, there is an urgent need for new antimalarial drugs, ideally with novel mechanisms of action.

Benzoxaboroles have shown potent activity against a wide range of infectious pathogens, including bacteria (3, 4), fungi (5), and protozoans (6–9). The highly electrophilic nature of the boron component of these compounds leads to interaction with a variety of protein targets (10, 11). Enzymes targeted in other systems have included leucyl-tRNA synthetase (4, 5), phosphodiesterase 4 (12), kinases (13), and β-lactamase (14). Previously studied benzoxaboroles with potent antimalarial activity have been shown to target leucyl-tRNA synthetase (8) and a cleavage and polyadenylation specificity factor (CPSF) homolog (15). CPSF homologs have also been the targets of benzoxaboroles acting against the protozoan pathogens Toxoplasma gondii (16) and Trypanosoma brucei (17, 18).

A robust pipeline of new antimalarial compounds is under development, and it is important to explore many chemical families (19). For active compounds with drug-like properties, it is very helpful to understand mechanisms of action and resistance. A valuable means of characterizing mechanisms is to study P. falciparum selected for resistance to compounds of interest (20). This report concerns characterization of mechanisms for AN13762, a benzoxaborole that was declared a preclinical candidate in 2017 but that was later withdrawn due to unexplained toxicity in animal trials. AN13762 remains a valuable compound for exploring mechanisms of action and resistance of antimalarial oxaboroles. Indeed, the compound appears to have a novel mechanism of antimalarial action and multiple mechanisms of resistance selected by increasing concentrations of the compound.

## RESULTS

### Antimalarial activity of AN13762 and related benzoxaboroles.

**FIG 1:**
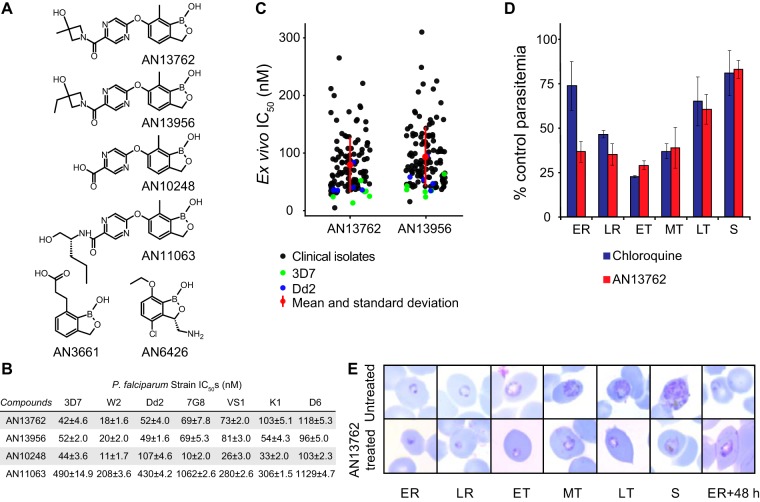
*In vitro* activity of benzoxaboroles against P. falciparum. (A) Chemical structures of benzoxaboroles. (B) Sensitivity of laboratory strains to benzoxaboroles. Values shown are mean IC_50_s ± standard deviation based on 3 independent assays per strain, each performed in triplicate. (C) Sensitivity of fresh P. falciparum isolates, collected in Tororo, Uganda, from 2016 to 2017, to benzoxaboroles. Each point represents a single assessment for a clinical isolate or control laboratory strain. Mean values and standard deviation are shown in red. (D) Stage specificity of action of AN13762. P. falciparum Dd2 was incubated with 0.5 μM AN13762, 1.3 μM chloroquine (each ∼10× the IC_50_), or 0.1% DMSO for 8-h intervals across the life cycle and then continued in culture until the following ring stage, when parasitemias were counted and compared. Error bars represent standard deviations from 3 independent experiments. (E) Morphology of parasites treated with 0.5 μM AN13762. Photomicrographs represent parasites treated with AN13762, beginning at the early ring stage, and untreated controls. ER, early ring; LR, late ring; ET, MT, and LT, early, midstage, and late trophozoites, respectively; S, schizonts; h, hours.

AN13762 was identified from a lead optimization program informed by the characterization of other benzoxaboroles and focused on achieving a single-dose treatment for falciparum malaria (8, 15, 21–23). AN13762 demonstrated rapid, nanomolar *in vitro* activity against multiple strains of P. falciparum, potent activity against Plasmodium berghei and P. falciparum in murine models, pharmacokinetics and bioavailability suggesting cure after a single oral dose, and acceptable safety signals to warrant establishment as a preclinical candidate by the Medicines for Malaria Venture (21). AN13762 and structurally related benzoxaboroles demonstrated similar potencies against laboratory strains and fresh Ugandan isolates of P. falciparum (Fig. 1). AN13762 was most active against ring- and trophozoite-stage parasites. Parasites treated with AN13762 had abnormal trophozoite morphology and did not develop beyond this stage.

### Selection and analysis of parasites resistant to AN13762 and related benzoxaboroles.

**FIG 2:**
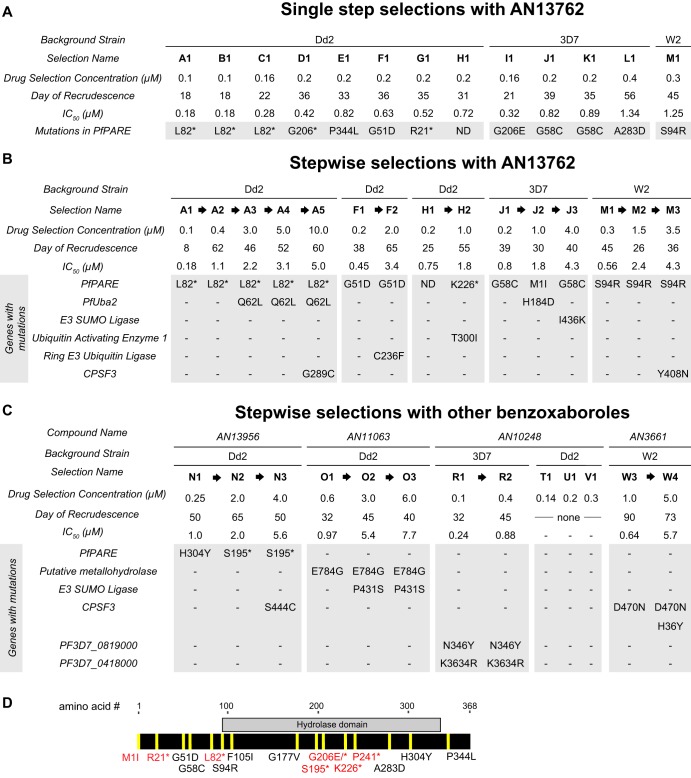
Schematics showing resistance selections and SNPs identified in selected parasites (shaded). Stop codons are indicated by asterisks. (A) Single-step selections with AN13762 for indicated strains. We observed no dominant mutation (>60% mutant reads at position) in strains D1, E1, F1, and G1. Upon closer examination, several low-prevalence (20 to 50%) mutations were identified in PfPARE, with the most abundant mutation noted (Data Set S1). (B) Stepwise selection with AN13762. In strain H2, a single nucleotide insertion (435529_435530) and subsequent frameshift leading to a premature stop codon was observed. (C) Stepwise selection with other benzoxaboroles. For selections T1, U1, and V1, parasites did not recrudesce over 90 days. The results for AN3661-resistant strains W3 and W4 were published previously (Sonoiki et al. [15]). (D) Map of nonsynonymous mutations selected in PfPARE.

To gain insights into mechanisms of action and resistance, we cultured P. falciparum with AN13762 to select, in a single step or stepwise manner, for parasites with decreased sensitivity. For several independent single-step selections, parasites were initially undetectable on Giemsa-stained smears, followed by regrowth. With 100 to 200 nM AN13762, parasites regrew after 18 to 39 days; with greater concentrations, regrowth occurred more slowly (Fig. 2A). In stepwise selections, greater degrees of resistance were also selected more slowly (Fig. 2B). Genomic DNA was extracted from parasites after each step of selection and analyzed by whole-genome sequencing. All sequence data are described in Data Sets S1 and S2 in the supplemental material. Notably, all parasites selected for decreased sensitivity to AN13762 contained single-nucleotide polymorphisms (SNPs) in PF3D7_0709700, which encodes a lysophospholipase homolog recently named prodrug activation and resistance esterase (PfPARE) due to its demonstrated activation of antimalarial esters (24). Parasites with multiple different PfPARE SNPs, including nonsense mutations, were selected (Fig. 2D). After additional selection with higher concentrations of AN13762, most parasites contained, in addition to PfPARE mutations, SNPs in enzymes predicted to function within ubiquitination or sumoylation pathways, specifically, SUMO-activating enzyme subunit 2 (PfUba2; Pf3D7123700, lines A3 to A5), E3 SUMO ligase (Pf3D71360700, line J3), ring zinc finger E3 ubiquitin ligase (Pf3D7_0529900, line F2), and ubiquitin-activating enzyme 1 (Pf3D71350400, line H2). Two lines selected for very high-level resistance (A5 and M3) contained SNPs in a P. falciparum CPSF homolog (PfCPSF3) which was recently shown to be the target of another oxaborole antimalarial, AN3661 (15) (Fig. 2B). AN13762-resistant parasites did not demonstrate copy number amplification for any genes. After selection with AN13762, Dd2 and 3D7 lines with medium- to high-level resistance (A2 to A4, J2, and J3) were cultured without drug pressure. In each case, parasites cultured without drug pressure for 3 months had a stable phenotype, without notable change in sensitivity to AN13762 (Fig. S1).

10.1128/mBio.02640-19.1 FIG S1 Sensitivities of parasites selected for resistance to AN13762 after 15 to 90 days of culture without AN13762. Error bars represent standard deviations of the results from 3 independent experiments, each performed in triplicate. Download FIG S1, EPS file, 0.7 MB. Copyright © 2020 Sindhe et al. 2020 Sindhe et al. This content is distributed under the terms of the Creative Commons Attribution 4.0 International license.

10.1128/mBio.02640-19.4 DATA SET S1 Detailed SNP analysis of low-level benzoxaborole-resistant strains (separate file). Each tab represents an independently selected resistant strain. In strains D1, E1, F1, and G1 where there was no single dominant PfPARE mutation, we looked for mutations at lower proportion down to 0.2 mutant fraction. Download Data Set S1, XLSX file, 0.1 MB. Copyright © 2020 Sindhe et al. 2020 Sindhe et al. This content is distributed under the terms of the Creative Commons Attribution 4.0 International license.

Parasites were also selected for resistance to structural analogs of AN13762 with stepwise increases in concentrations of the compounds (Fig. 2C). For selection with AN13956, low-level-resistant lines (N1 and N2) had SNPs in PfPARE, and lines selected for higher-level resistance (N3) had an additional SNP in PfCPSF3, as observed for some lines selected with AN13762. For selection with AN11063, low-level-resistant parasites (O1) did not have SNPs in PfPARE but rather in a putative metallo-hydrolase. Parasites with higher-level resistance to AN11063 (O2 and O3) had an SNP in E3 SUMO ligase (Pf3D71360700) (Fig. 2C); another mutation in this gene was selected by AN13762 (J3). For selection with AN10248, Dd2 strain parasites did not emerge after incubation for 60 to 90 days with 140 to 300 nM compound. However, 3D7 strain parasites were selected for modest resistance after incubation with 100 nM AN10248 for 33 days and then 400 nM AN10248 for 28 days. Sequencing of the AN10248-resistant parasites revealed SNPs in two P. falciparum genes predicted to encode proteins of unknown function (PF3D7_0819000 and PF3D7_0418000). Copy number amplification was not detected for any parasites selected for resistance to AN13956, AN11063, or AN10248.

### PfPARE sequences of laboratory and field strains of P. falciparum.

In laboratory isolates, the PfPARE sequences are identical for the 3D7, Dd2, and 7G8 strains, except for the S238I polymorphism in 7G8. For field isolates, we sequenced PfPARE from 12 Ugandan isolates, 3 with relatively low (<50 nM), 5 with moderate (50 to 100 nM), and 4 with high (>100 nM) IC_50_s. Four SNPs were seen in these isolates, M158I, K340R, V420I, and T438A, the first in 10 isolates, and the others in 1 to 2 each, either as pure or mixed populations. For the common M158I mutation, the two isolates with wild-type (WT) sequences were two of three with relatively low IC_50_s, possibly identifying this mutation as a mediator of sensitivity to AN13762. None of the identified mutations in laboratory or field strains were those selected by culture with AN13762 (Fig. 2).

### Mutations in PfPARE mediate AN13762 resistance.

**FIG 3:**
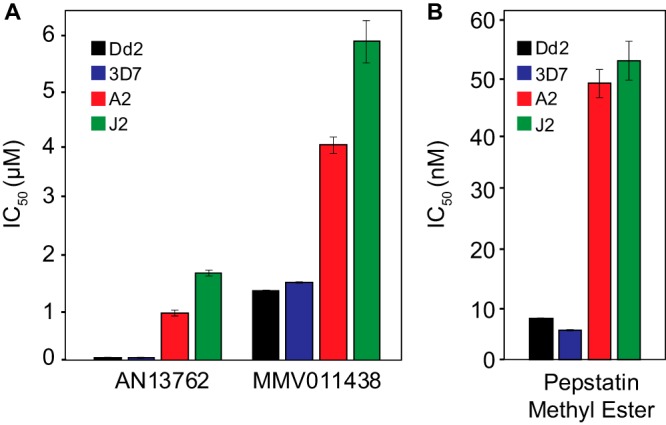
Sensitivities of wild-type (Dd2 and 3D7) parasites and those selected for resistance to AN13762 (A2 and J2) to AN13762 and two antimalarial esters. Error bars represent standard deviations of the results from 3 independent experiments, each performed in triplicate.

**FIG 4:**
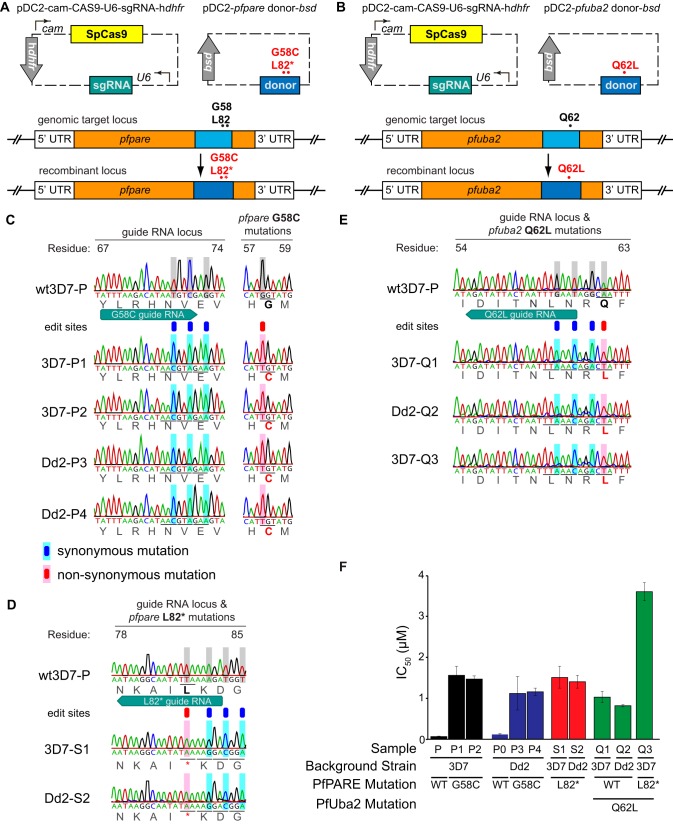
(A and B) CRISPR-Cas9-mediated gene editing. Dd2 and 3D7 parental parasites were transfected with a plasmid encoding the sgRNA, Cas9 nuclease, and human dihydrofolate reductase (h*dhfr*) as a selectable marker, and another plasmid encoding a donor sequence containing PfPARE G58C or L82* (A) and PfUba2 Q62L (B). (C to E) Electropherograms showing unmodified and genome-edited parasites. Gray boxes highlight nucleotides that differ from wild-type parasites. PfPARE and PfUBA2 single-letter amino acid substitutions and/or stop mutations are indicated in red. (F) Susceptibility to AN13762 of the parental (WT) lines and genetically modified lines. Lines P and P0 are WT strains. P1, P3, and Q1 were cultured under 150 nM AN13762 drug pressure, and lines P2, P4, Q2, Q3, S1, and S2 were selected with WR29910 and blasticidin. Bars represent mean IC_50_ values, and error bars are the standard deviations of the results from 3 independent experiments, each performed in triplicate. UTR, untranslated region.

Interestingly, PfPARE mutations have previously been reported to confer resistance in P. falciparum to antimalarial esters, both a series of pepstatin esters and MMV011438, presumably due to a requirement for esterase activity of PfPARE for antimalarial activity (24). Although AN13762 is not an ester, our characterization of resistant parasites suggested that this compound also requires activation by PfPARE for maximal activity. Consistent with this hypothesis, parasites selected for resistance to AN13762 were also resistant to pepstatin methyl ester and MMV011438 (Fig. 3). To further test the hypothesis that mutations in PfPARE mediate resistance to AN13762, we used CRISPR-Cas9 editing to engineer 3D7 and Dd2 parasites with the G58C or L82* PfPARE mutation (Fig. 4A). All transfectants demonstrated 100% editing efficiency at the appropriate sites, including engineered silent mutations at the single-guide RNA (sgRNA)-directed Cas9 binding sites. The PfPARE G58C and L82* mutations conferred a 10 to 30-fold decrease in AN13762 sensitivity, similar to changes in sensitivity in parasites that acquired these mutations after *in vitro* resistance selection (Fig. 4F). These results confirm a primary role for PfPARE mutations in conferring resistance to AN13762 and suggest that PfPARE-mediated activation of AN13762 is required for maximal antiparasitic activity.

### A mutation in PfUba2 mediates high-level AN13762 resistance.

Sequencing of parasites selected for high-level resistance to AN13762 identified both 3D7 and Dd2 strain parasites with mutations in PfUba2. To confirm that PfUba2 mutations mediate AN13762 resistance, we used CRISPR-Cas9 editing to engineer 3D7, Dd2, and PfPARE L82* parasites with the Q62L PfUba2 mutation (Fig. 4B). All transfectants demonstrated 100% editing efficiency at the appropriate sites, including engineered silent mutations at the sgRNA-directed Cas9 binding sites. In the engineered parasites, PfUba2 Q62L conferred a 30- to 60-fold decrease in AN13762 sensitivity (Fig. 4F). Transfectants that harbored both PfPARE L82* and PfUba2 Q62L mutations had an additional 40-fold decrease in AN13762 sensitivity, similar to sensitivities of parasites (lines A3 and A4) selected stepwise for high-level resistance and containing both of these mutations (Fig. 2B). These results confirm that PfUba2 mutations confer AN13762 resistance and that PfPARE and PfUba2 mutations have additive impacts on sensitivity to the compound.

### PfPARE processing of AN13762 is required for maximal antimalarial activity.

**FIG 5:**
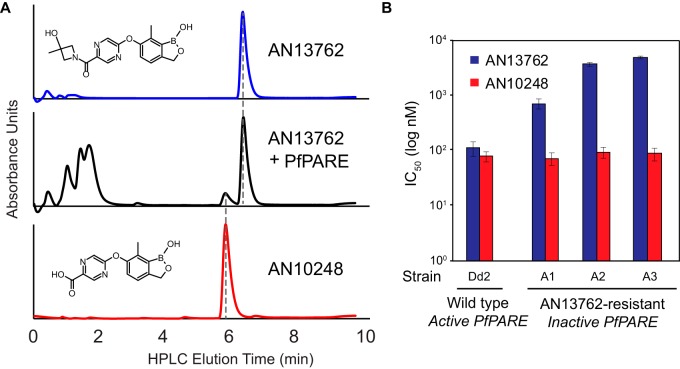
PfPARE processes AN13762 into AN10248. (A) LC-MS analysis of AN13762, AN13762 plus 0.1 mg PfPARE (incubated for 48 h), and AN10248. The structures of the two compounds are shown. (B) Activities of AN13762 and AN10248 against P. falciparum strains with wild-type and PfPARE-inactivated parasites; values are based on 3 independent experiments, each performed in triplicate. HPLC, high-performance liquid chromatography.

The results described above suggest that AN13762 is activated in parasites to a more active metabolite by PfPARE. To test this hypothesis, we expressed recombinant PfPARE in Escherichia coli. Recombinant PfPARE demonstrated esterase activity, with hydrolysis of *p*-nitrophenyl butyrate (PNPB) to free *p*-nitrophenol (PNP) (Fig. S2). When recombinant PfPARE was incubated with AN13762, we observed formation of AN10248 (Fig. 5A). Thus, PfPARE hydrolyzes AN13762 intracellularly to the active antimalarial AN10248, and loss of the ability to activate the parent compound leads to decreased AN13762 sensitivity. Of interest, PfPARE mutations that led to decreased sensitivity to AN13762 did not notably alter sensitivity to AN10248 (Fig. 5B).

10.1128/mBio.02640-19.2 FIG S2 PfPARE expression, purification, and hydrolase activity. Recombinant PfPARE was expressed in E. coli and purified with an Ni-NTA column, as described in Materials and Methods. (a) Purification. The lanes show MW standards, unpurified recombinant protein stained with Coomassie blue, and a Western blot probed with anti-His primary antibody. Recombinant PfPARE is marked with an arrow. (b) Esterase assay. Production of the PNP product (indicated as the absorbance at 405 nm) is shown over time for reactions with different concentrations of the PNPB starting material. Download FIG S2, EPS file, 1.7 MB. Copyright © 2020 Sindhe et al. 2020 Sindhe et al. This content is distributed under the terms of the Creative Commons Attribution 4.0 International license.

### AN13762 appears to exhibit a novel antimalarial mechanism.

**FIG 6:**
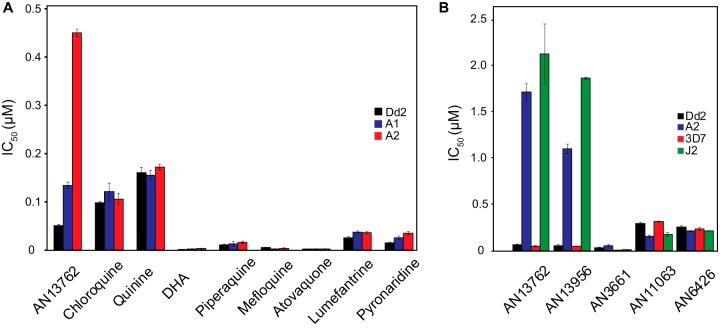
Sensitivities of wild-type and benzoxaborole-resistant parasites. (A and B) Sensitivities of wild-type parasites and those selected for resistance to AN13762 are shown for standard antimalarials (A) and benzoxaboroles (B). Error bars represent standard deviations of the results from 3 independent experiments. DHA, dihydroartemisinin.

**FIG 7:**
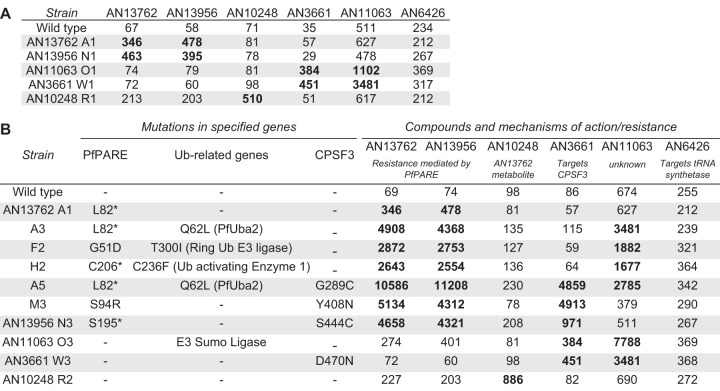
(A and B) Sensitivities (IC_50_, in nanomolar) of low-level (A) and high-level (B) benzoxaborole-resistant parasites to all tested benzoxaboroles. Ub, ubiquitin.

Selection for AN13762 resistance identified complex resistance mechanisms but did not clearly point to a biochemical target. To gain insight into mechanisms of action, we assessed cross-resistance between AN13762, related compounds, other benzoxaboroles with known antimalarial mechanisms, and a panel of standard antimalarial drugs. Parasites selected for moderate resistance to AN13762 did not have markedly altered sensitivity to any of a panel of 8 standard antimalarial drugs (Fig. 6A) or to other benzoxaboroles, including those known to target P. falciparum PfCPSF3 (AN3661) (15) and leucyl-tRNA synthetase (AN6426) (8) (Fig. 6B). Cross-resistance was seen between AN13762 and AN13956. We then compared the sensitivities of parasites selected independently for resistance to 5 different benzoxaboroles. For parasites selected for moderate resistance, cross-resistance was seen between AN13762 and AN13956 and between AN3661 and AN11063 (Fig. 7A). Considering parasites selected for higher levels of resistance, cross-resistance was seen consistently between AN13762 and AN13956 at all levels of resistance, between AN13762 and AN11063 with some, but not other, mutations, and between AN13762, AN13956, and AN3661 for parasites selected for mutations in PfCPSF3 (Fig. 7B). Cross-resistance was consistent with the selection of parasite genotypes. Parasites selected for resistance to AN10248, the active metabolite of AN13762, were not cross-resistant with any other compounds, and parasites selected for resistance to 5 other benzoxaboroles were not resistant to the leucyl-tRNA synthetase inhibitor AN6426.

We also compared the sensitivities of wild-type 3D7 strain parasites and those selected for resistance to AN13762 to the components of the MMV Malaria Box (387 compounds) and Pathogen Box (127 compounds), collections of compounds previously shown to be active against P. falciparum or other pathogens (https://www.mmv.org/mmv-open/pathogen-box) (25). In screening with 3× the IC_50_ for each compound, only one compound showed cross-resistance with AN13762-resistant strains A2 and J2 (Data Set S3). This compound was MMV011438, the antimalarial ester for which selection of resistance was accompanied by mutations in PfPARE (24).

10.1128/mBio.02640-19.5 DATA SET S2 Detailed SNP analysis of high-level benzoxaborole-resistant strains (separate file). Each tab represents an independently selected resistant strain. In strain H2, a single nucleotide insertion (435529_435530insT) and subsequent frameshift leading to a premature stop codon were observed. Download Data Set S2, XLSX file, 0.4 MB. Copyright © 2020 Sindhe et al. 2020 Sindhe et al. This content is distributed under the terms of the Creative Commons Attribution 4.0 International license.

10.1128/mBio.02640-19.6 DATA SET S3 IC_50_ values for wild-type and AN13762-resistant strains (A2 and J2) are listed for compounds from the Malaria Box and Pathogen Box (separate file). Only compound MMV011438 from plate E of the Malaria Box showed cross-resistance to AN13762 (only 40% inhibition was seen in both the Dd2 and 3D7 strains). Hence, we used 40% inhibition as a threshold to consider cross-resistance of the compounds to AN13762-resistant strains. None of the compounds from the Pathogen Box showed inhibition in both resistant strains. Download Data Set S3, XLSX file, 0.3 MB. Copyright © 2020 Sindhe et al. 2020 Sindhe et al. This content is distributed under the terms of the Creative Commons Attribution 4.0 International license.

## DISCUSSION

Benzoxaboroles have shown promising antimalarial activity, with different benzoxaboroles acting against different P. falciparum targets (8, 15). Most recently, AN13762 demonstrated improved drug-like properties compared to other benzoxaboroles, including excellent *in vitro* and *in vivo* potency against P. falciparum, leading to its declaration as a preclinical candidate by the Medicines for Malaria Venture (21). Although development of the compound was later halted due to unexplained animal toxicity, it remains of interest for the characterization of antimalarial mechanisms of action and resistance. Selection and characterization of P. falciparum with decreased sensitivity to AN13762 identified a complex mechanism of resistance, with loss of function of PfPARE associated with low-level resistance, and mutations in five other proteins, most participating in ubiquitin/sumoylation enzyme cascades, associated with high-level resistance. These selections did not identify a single P. falciparum target for AN13762, but cross-resistance studies suggested a mechanism of action unique from that of other studied benzoxaboroles.

The characterization of sequences of P. falciparum selected for resistance *in vitro* has offered valuable insights into mechanisms of action and resistance, including the identification of dozens of independent targets for recently characterized antimalarial compounds (20). In the case of benzoxaboroles, studies showed AN6426 and AN8432 to target P. falciparum leucyl-tRNA synthetase (8) and AN3661 to target PfCPSF3, a homolog of a component of the mammalian CPSF complex (15). We embarked on a similar strategy to characterize mechanisms for AN13762. Midnanomolar concentrations of AN13762 readily selected for parasites with low-level resistance (midnanomolar IC_50_s against cultured parasites). Higher-level resistance (micromolar IC_50_s) was subsequently selected by micromolar concentrations of AN13762, although longer-duration incubations were required to select high-level resistance.

Parasites selected for low-level resistance to AN13762 consistently contained any of a number of mutations, including null mutations, in PfPARE, a lysophospholipase homolog for which mutations were also recently shown to mediate resistance to the antimalarial compound MMV011438 and a series of pepstatin esters (24). Incubation of P. falciparum with MMV011438 and pepstatin butyl ester selected for mutations in PfPARE, and it was shown that PfPARE acted as an esterase to activate these compounds. AN13762 is not an ester, and this parent compound has potent activity, but our results suggest that it too requires activation by PfPARE for maximal antimalarial effects. Specifically, we demonstrated that recombinant PfPARE hydrolyzed AN13762 to AN10248, a compound that is also a potent antimalarial, and that introduction of a PfPARE null mutation yielded similar levels of resistance to those in parasites selected with midnanomolar concentrations of AN13762. Taken together, our results strongly suggest that intracellular hydrolysis of AN13762 to AN10248 by PfPARE is required for full activity. We hypothesize that intracellular access of AN10248, a carboxylic acid, is limited, and that intracellular activation of AN13762 to AN10248 enables a more potent antimalarial effect. Other benzoxaboroles similarly showed antitrypanosomal activity based on intracellular activation of prodrugs (26).

Additional insight into resistance mechanisms was gained by selecting for higher-level resistance with micromolar concentrations of AN13762. These selections yielded parasites with high-level resistance (IC_50_s of >1 μM against cultured parasites). These resistant parasites contained PfPARE mutations plus additional mutations in any of 5 different genes. Interestingly, 4 of the additional genes mutated in highly resistant parasites encode components of P. falciparum ubiquitin or sumoylation cascades. More specifically, SUMO-activating enzyme subunit 2 was mutated in resistant 3D7 and Dd2 strain P. falciparum, with IC_50_s of 2.2 μM or higher, E3 SUMO ligase was mutated in 3D7 strain parasites, with an IC_50_ of 4.3 μM, ubiquitin-activating enzyme 1 was mutated in Dd2 strain parasites, with an IC_50_ of 1.8 μM, and E3 ubiquitin ligase was mutated in Dd2 strain parasites, with an IC_50_ of 3.4 μM. CRISPR-Cas9-mediated introduction of PfPARE and PfUba2 mutations recapitulated resistance to AN13762, confirming their mediation of altered parasite sensitivity. Mutations in PfPARE and PfUba2 had additive effects in conferring resistance to AN13762, with the degree of resistance similar in double mutants either engineered by CRISPR-Cas9 mutagenesis (3.7 μM) or selected by culture with stepwise increasing concentrations of AN13762 (2.2 to 3.1 μM).

Ubiquitination and sumoylation pathways participate in posttranslational modifications to label proteins for proteasomal degradation and regulate a range of cellular processes, and both pathways participate in cellular stress responses. In P. falciparum, ubiquitination (27) and sumoylation (28, 29) appear to serve as important regulators of stress responses. The stress response engendered by treatment with artemisinins includes an accumulation of ubiquitinated proteins (30). Synergism was seen between artemisinins and proteasome inhibitors, supporting the P. falciparum stress response as a target for artemisinins. Accumulation of ubiquitinated proteins was diminished in artemisinin-resistant parasites, suggesting an enhanced stress response in resistant parasites (30). Our results showed that high-level resistance to AN13762 was associated with mutations predicted to disrupt the ubiquitination and sumoylation systems, suggesting that, in contrast to results with artemisinins, a diminished stress response facilitated drug resistance. Presumably, AN13762 exerts antimalarial activity in part by augmenting stress responses, and alterations in these responses, via mutations in at least 4 different ubiquitination/sumoylation enzymes, enable high-level drug resistance. These results further indicate that mutations altering ubiquitination/sumoylation enzymes are tolerated by malaria parasites. Some of these mutant parasites were shown to be stable in culture without drug pressure, but the overall impacts of the mutations on parasite fitness are not known.

Resistance to the related benzoxaborole AN11063 was associated with a mutation in a predicted metallo-hydrolase. In a situation analogous to that for AN13762, low-level resistance (IC_50_, 0.98 μM) to AN11063 was associated with the metallo-hydrolase mutation, and higher-level resistance was associated with the addition of a mutation in E3 SUMO ligase (IC_50_, 5.4 μM). We hypothesize that the metallo-hydrolase metabolizes AN11063 to a more potent intracellular compound, as is the case with PfPARE and AN13762. Interestingly, resistance to AN10248, the active metabolite of AN13762, was not readily selected, although for 3D7 (but not Dd2) parasites, resistant strains with mutations in two poorly annotated parasite genes were identified.

Some selections of high-level resistance to both AN13762 (IC_50_, 4.3 μM in W2 strain parasites) and AN13956 (IC_50_, 5.6 μM in Dd2 strain parasites) were accompanied by mutations in both PfPARE and PfCPSF3. PfCPSF3 is a homolog of a component of the mammalian CPSF complex, which is required for maturation of mRNAs, and was discovered after another benzoxaborole, AN3661, selected for mutations in this protein (15). PfCPSF3 was shown to be the P. falciparum target for AN3661, as incubation with the compound led to loss of transcripts for trophozoite proteins in treated wild-type parasites, as expected with loss of CPSF activity, but not in treated mutant parasites. Our new results indicating that high-level resistance to AN13762 and AN13956 can also be accompanied by mutations in PfCPSF3 suggest that this protein is also a target for these other benzoxaboroles. However, selection of PfCPSF3 mutations only in highly resistant parasites and the lack of cross-resistance between AN13762 and AN3661 (except for highly resistant parasites with PfCPSF3 mutations) suggest that PfCPSF3 is not the primary target of AN13762. Taken together, our results from prior studies (8, 15) and those reported here suggest that benzoxaboroles target multiple proteins in P. falciparum, complicating drug discovery for this class of antimalarials.

Our study has important limitations. First, it is not clear that the multiple mutations identified as contributing to resistance fully explain the activity of AN13762. It is noteworthy that activity against laboratory and field strains varied quite widely; this variation is not fully explained by the identified mutations. Second, although our genetic evidence was solid and we showed hydrolysis of AN13762 to AN10248 *in vitro*, technical challenges limited our ability to biochemically confirm this metabolism in intact parasites. Specifically, we were unable to detect AN10248 in cultured parasites treated with AN13762. Third, although we identified mutations in multiple genes that encode members of ubiquitination and sumoylation pathways in resistant parasites, we were unable to identify changes in ubiquitination or sumoylation in the mutant parasites. Fourth, although metabolism by PfPARE of AN13762 to AN10248 was required for full activity, the two compounds had similar antimalarial activities; we predict that AN10248 has better access to intracellular drug targets, but this has not been proven.

Consideration of cross-resistance in parasites selected for resistance to benzoxaboroles was informative. AN13762 and AN13956 were cross-resistant, consistent with shared mechanisms of action. Cross-resistance was not seen between AN13762 and other benzoxaboroles known to target leucyl-tRNA-synthetase. Cross-resistance was seen with AN3661, which targets PfCPSF3, but only with highly resistant parasites that had mutated PfCPSF3. AN13762 was also not cross-resistant with standard antimalarials, with similar activities against parasites sensitive or resistant to chloroquine and with minimal differences in activities of 8 tested antimalarials against parasites sensitive or highly resistant to AN13762. Further, in a screen of 514 compounds included in the MMV Malaria Box and Pathogen Box, the only compound that showed clear cross-resistance with AN13762 was MMV011438, the ester that, as explained above, also selects for resistance via mutation of PfPARE. Taken together, these results suggest a unique set of mechanisms of resistance and quite likely a novel antimalarial mechanism of action for AN13762 and closely related benzoxaboroles.

## MATERIALS AND METHODS

### Culture of malarial parasites.

Human red blood cells infected with P. falciparum were cultured using standard methods at 2% hematocrit in RPMI 1640 medium (Invitrogen) supplemented with 0.5% AlbuMAX II (Gibco Life Technologies), 2 mM l-glutamine, 100 mM hypoxanthine, 5 μg/ml gentamicin, 28 mM NaHCO_3_, and 25 mM HEPES at 37°C in an atmosphere of 5% O_2_, 5% CO_2_, and 90% N_2_. P. falciparum strains were obtained from the Malaria Research and Reference Reagent Resource Center, Manassas, Virginia.

### Activity against cultured laboratory strains of P. falciparum.

The studied benzoxaboroles were analyzed by nuclear magnetic resonance, liquid chromatography-mass spectrometry, and high-performance liquid chromatography for structural characterization and purity. The studied antimalarials were from Sigma-Aldrich, except for piperaquine, which was from Jinan Jiaquan International Trade Co. Pepstatin methyl ester was from EMD Millipore, and MMV011438 was a gift from Daniel Goldberg. Parasites were synchronized by treatment with 5% d-sorbitol and cultured in triplicate 96-well culture plates (200 μl per well) with serially diluted compounds at concentrations of 1 nM to 10 μM, with a total of 8 concentrations tested for each compound, and final dilutions containing 0.2% dimethyl sulfoxide (DMSO). Compounds from the MMV Malaria Box (387 compounds, a gift from Robert St. Onge) and Pathogen Box (127 compounds, acquired from MMV) were screened against 3D7 and Dd2 strain parasites and strains selected for resistance to AN13762 (A2 and J2) at 3× the IC_50,_ determined against the wild-type strains; AN13762 was included as a control. After 48 h of incubation with test compounds, cultures were fixed with 2% formaldehyde for 24 h at 37°C or 48 h at room temperature, cells were stained with 4 nM YOYO-1 dye (Molecular Probes), and the counts of treated and control cultures were determined with a fluorescence-activated cell sorter. IC_50_s were calculated by nonlinear regression using the GraphPad Prism software.

### Activity against fresh P. falciparum field isolates.

The activities of benzoxaboroles were tested against fresh P. falciparum isolates using a 72-h growth inhibition assay with Sybr green detection, as previously described (31). Isolates were collected from March 2016 to July 2017 from patients in the Tororo and Busia Districts, Uganda, who were newly diagnosed with P. falciparum malaria; samples were collected before antimalarial treatment was administered. The relevant clinical trials (registered at ClinicalTrials.gov no. NCT02163447) and analyses of cultured parasites were approved by the Uganda National Council of Science and Technology, the Makerere University Research and Ethics Committee, and the University of California, San Francisco Committee on Human Research. Informed consent was obtained from blood donors or their parents/guardians.

### Stage specificity and morphological effects of AN13762.

The stage-specific activities of AN13762 and chloroquine were analyzed as previously described (32). Highly synchronous Dd2 P. falciparum cells (synchronized by treatment with 5% d-sorbitol) were cultured in triplicate in 96-well culture plates with 500 nM AN13762 or 1.3 μM chloroquine for 8-h intervals, beginning at the ring stage; each culture was incubated with the test compound for only one 8-h interval within the parasite life cycle. Control cultures contained equivalent concentrations of DMSO (0.2%). At the end of each interval, the cultures were washed three times and resuspended in culture medium without drug. After 48 h, when control parasites were at the ring stage, the cultures were fixed with 2% formaldehyde and counted using flow cytometry, as detailed above, and parasitemias were compared with those of untreated control parasites. Giemsa-stained smears of parasites incubated with AN13762 for different intervals were examined microscopically and photographed.

### Selection of parasites with decreased sensitivity to benzoxaboroles.

Triplicate 10-ml cultures of 3D7, Dd2, and W2 strains of P. falciparum, each containing a clonal population of 6 × 10^7^ asynchronous parasites, were incubated with benzoxaboroles. In single-step selections, parasites were incubated with 100 to 400 nM compound. In stepwise selections, parasites were incubated with increasing concentrations, as outlined in Fig. 2. In all selections, cultures were monitored with Giemsa-stained smears. At each step of selection, parasites were initially undetectable, followed by regrowth. When parasites achieved multiplication rates approximately equivalent to those of untreated controls, IC_50_s were determined, and aliquots were frozen for long-term storage. For stepwise selections, parasites were incubated with increased concentrations of compounds, as detailed in Fig. 2. Resistant strains A2, A3, and A4 were grown without drug pressure for up to 90 days, with regular assessment of sensitivity to AN13762. Mutant clones with altered sensitivity to test compounds will be made available to researchers through the Malaria Research and Reference Reagent Resource Center (MR4) upon request.

### Whole-genome sequencing.

Genomic DNA was extracted from parasite cultures (10 ml at 2% hematocrit and ∼10% parasitemia) using the PureLink genomic DNA kit (Invitrogen) and extracted per manufacturer’s instructions. Paired-end next-generation sequencing libraries were constructed from the genomic DNA (gDNA) using the NEBNext Ultra II kit (New England BioLabs). The gDNA (200 ng) was enzymatically fragmented for 17.5 min at 37°C, and the reaction was quenched with EDTA. The samples were cleaned with AMPure beads (Beckman Coulter) at a sample-to-bead ratio of 1:1.4 and eluted in sterile H_2_O. Eluted samples were run on a Bioanalyzer high-sensitivity (HS) DNA kit (Agilent) to confirm the presence of 200- to 300-bp gDNA fragments. Samples were then subjected to end prep and adaptor ligation using a 1:10 dilution of the NEBNext adaptor, all as described in the NEBNext Ultra II protocol. After digestion with the USER enzyme, a second AMPure bead cleanup was carried out with a sample-to-bead ratio of 1:0.9 for 15 min prior to placing the beads on a magnet and eluting with sterile H_2_O. Samples were then indexed with 9 cycles of PCR using NEB Q5 polymerase, unique TruSeq i5/i7 barcode primers, and conditions according to the NEB Q5 protocol. A final AMPure bead cleanup step was performed at a sample-to-bead ratio of 1:0.9, with elution with sterile H_2_O. Library quality was assessed with a Bioanalyzer HS DNA kit, with broad peaks from 200 to 500 bp observed in each sample, as expected. Libraries were pooled and sequenced on an Illumina HiSeq 4000 platform using a PE100 (paired end 100 base pair) flow cell.

### Sequence analysis.

After demultiplexing, fastq files were filtered with PriceSeqFilter (33) with the flags “-rqf 100 0.99 -rnf 100,” specifying that in all reads, 100% of nucleotides must have a 99% chance of being correct, and that no reads may contain any ambiguous characters. The filtered fastq files were then aligned to the 3D7 genome from PlasmoDB, version 26 (34), using Bowtie2 (35), with the following flags: “-p 20 –very-sensitive -x.” The resulting SAM files were imported into Geneious version 10.0.9 (36) and run through MinorityReport to assess genetic differences (37). The aligned sequencing reads for genes of interest were input along with the FASTA and GFF files for the 3D7 reference genome from PlasmoDB, version 26 (34). The thresholding flags “-wtc 5,” “-vp 0.6,” and “-vc 5” were used, requiring each reported variant to have a coverage of at least 5 reads in both the parent and the mutant and at least 60% of the mutant reads to contain the variant. Gene copy number variation analysis was performed to calculate the ratio of reads that map to each sliding window in the mutant and parent data sets, normalized by the total number of reads for the parent and mutant (37).

### CRISPR-Cas9-mediated editing of PfPARE and PfUba2 mutations.

Choice of sgRNA, plasmid construction, and parasite transfections were performed as described previously (38). The primers used for sgRNA and donor constructs are listed in Table S1. G58C and L82* mutations in PfPARE and the Q62L mutation in PfUba2 were chosen for CRISPR-Cas9 engineering in wild-type parasites. sgRNAs were inserted into a pDC2-cam-Cas9-U6-hdhfr plasmid that encodes Streptococcus pyogenes Cas9 expressed under the calmodulin promoter and a human dihydrofolate reductase (dhfr) selectable marker (that mediates resistance to the human dhfr inhibitor WR99210); the sgRNA was expressed with a U6 promoter. A 1.2-kb fragment of donor template was amplified from wild-type (3D7 and Dd2) and AN13762-resistant (A2 and J2) strains containing the mutation of interest. Using the QuikChange Lightning multisite-directed mutagenesis kit, 3 or 9 nucleotide substitutions were introduced into the donor sequences at sgRNA-binding sites to protect from further Cas9 recognition and cleavage (binding site mutations are detailed in Table S1). The resulting fragment was inserted using the ApaI and BamHI restriction sites into the pDC2-bsd plasmid, which expresses the blasticidin *S*-deaminase (bsd) selectable marker. Each of the pDC2-PfPARE donor-bsd plasmids encoding PfPARE wild-type and G58C mutants was transfected 2 times into Dd2 parasites and 2 times into 3D7 parasites by electroporation, along with the pDC2-cam-Cas9-U6-sgRNA-hdhfr plasmid. Of those 4 transfections per plasmid, two were maintained in 2.5 nM WR99210 (Sigma-Aldrich) and 2 mg/ml blasticidin (Sigma-Aldrich), and another two were maintained in 150 nM AN13762. Similarly, transfections were carried out for PfUba2 mutant (Q62L) parasites in 3D7 and Dd2 and in a 3D7 parasite carrying both the PfPARE (L82*) and PfUba2 (Q62L) mutations. Transfections were performed in 400 μl of 1× Cytomix containing 10% rings and 100 μg of Cas9 and donor plasmids per transfection. Electroporation was performed in a cuvette at 0.310 kV and 950 μF. Transfectants were maintained under standard culture conditions for 4 weeks, and then gDNA was extracted. Fragments of PfPARE and PfUba2 were amplified from gDNA using Phusion high-fidelity DNA polymerase (Thermo Scientific), and the primers are described in Table S1. The PCR conditions used were 98°C for 5 min; 30 cycles of 98°C for 10 s, 60°C for 20 s, and 72°C for 80 s; and 72°C for 5 min. The amplified fragments were cleaned using AMPure beads (Beckman Coulter) and Sanger sequenced at Eurofins Genomics. IC_50_ values were obtained for all transfectants using the methods described above.

10.1128/mBio.02640-19.3 TABLE S1 List of primers used for cloning and CRIPSR-Cas9 editing of genes. Download Table S1, DOCX file, 0.1 MB. Copyright © 2020 Sindhe et al. 2020 Sindhe et al. This content is distributed under the terms of the Creative Commons Attribution 4.0 International license.

### Recombinant protein expression and esterase assay.

The *PfPARE* gene (PF3D7_0709700) was amplified from genomic P. falciparum DNA using the primers PfPARE-ExP-F and PfPARE-ExP-R (Table S1). The gene was cloned into a pET-28b(+) plasmid between the NcoI and XhoI restriction sites with a TaKaRa In-Fusion cloning kit to obtain recombinant protein, with a C-terminal His6 tag with a molecular weight (MW) of 43,423 Da. The His-tagged PfPARE pET-28b construct was transformed into Rosetta 2(DE3) Singles E. coli competent cells (Novagen), and these cells were grown overnight and used to inoculate fresh cultures, which were grown for 2 to 4 h to optical density 0.5 to 0.7 and induced with 0.5 mM isopropyl-β-d-thiogalactopyranoside (IPTG) for 6 h at 30°C. The bacterial pellet was then lysed by sonication in 50 mM Tris-HCl (pH 8), 200 mM NaCl, 10% glycerol, 0.1% Triton X-100, and protease inhibitor cocktail (Roche cOmplete tablet). Supernatant containing 10 mM imidazole was incubated with 2 ml of 50% nickel-nitrilotriacetic acid (Ni-NTA) agarose beads (Qiagen) with rotation for 1 h at 4°C. The lysate was then allowed to flow through under gravity, and the column was washed twice with 25 ml of 20 mM imidazole. The recombinant His tag fusion protein was eluted with lysis buffer containing 250 mM imidazole. For assays with PNPB, 0.1 mg of purified protein was incubated with 0 to 833 mM PNPB (prepared as a 50 mM stock in acetonitrile) in phosphate-buffered saline (PBS) at 37°C, and reactions were monitored for absorbance at 405 nm, indicating formation of PNP using a Tecan Infinite 200 Pro plate reader.

### LC-MS analysis.

For assays analyzed by liquid chromatography-mass spectrometry (LC-MS), 0.1 mg of protein was incubated for 48 h at 22°C with 20 nmol compound in a 100-μl reaction mixture. Heat-denatured enzyme was used as a negative control. The reaction mixture was filtered using an Amicon Ultra centrifugal filter (0.5 ml, 3-kDa cutoff) to remove protein, and 20 to 50 μl flowthrough was injected on the LC-MS. The samples were analyzed on a Waters Micromass ZQ mass spectrometer equipped with a Waters 2795 separation module, Waters 2424 evaporative light scattering detector, and Waters 2996 photodiode array detector. Separations were carried out with an XTerra MS C_18_, 5-mm, 4.6- by 50-mm column at ambient temperature using a mobile phase of water-methanol containing a constant 0.1% formic acid.

### Data availability.

The raw data have been deposited under NCBI Sequence Read Archive number SRP228727 (https://trace.ncbi.nlm.nih.gov/Traces/sra/?study=SRP228727) and BioProject number PRJNA504044 (https://www.ncbi.nlm.nih.gov/bioproject/?term=PRJNA504044). This represents the minimal underlying data set for this study.
