# Supplementary material for: Plasmodium falciparum Resistance to a Lead Benzoxaborole Due to Blocked Compound Activation and Altered Ubiquitination or Sumoylation
Source: mBio. 2020 Jan 28;11(1):e02640-19. doi: 10.1128/mBio.02640-19 (PMC6989105; doi:10.1128/mBio.02640-19)
Supplement: TABLE S1 [file mBio.02640-19-st001.docx]

| **Primer Name** | **Primer sequence** |
| --- | --- |
| PfPARE-G58C-SgRNA-F | ATTGGATTTAAGACATAATGTCG |
| PfPARE-G58C-SgRNA-R | AAACCGACATTATGTCTTAAATC |
| PfPARE-L83*-SgRNA-F | ATTGGATAAGGCAATATTAAAAGA |
| PfPARE-L83*-SgRNA-R | AAACTCTTTTAATATTGCCTTATC |
| PfUba2-Q62L-SgRNA-F | ATTGGAGATATTACTAATTTGAAT |
| PfUba2-Q62L-SgRNA-R | AAACATTCAAATTAGTAATATCTC |
| PfUba2-Apa1-F | AGCGGGCCCATGCATAAAACGATAAGAAAGCTATTTAGCG |
| PfUba2-BamH1-R | GATCGGATCCGTGACGATAACATGAGTACATTGTCATC |
| PfPARE-Apa1-F | AGCGGGCCCATGAAGAGCCAGGGTGGAGGGAAGATATCG |
| PfPARE-BamH1-R | GATCGGATCCTTATACTTGTTCTTCTTGTTTGGGGGTATGGACAGC |
| PfPARE_L83SitedirectedM-F | CTCCTCAATCCAACTATTTTTATATATATAATAATTATCTCCGTCCTTTTATATTGCCTTATTATTATTTACTACCTCGACATTATGTC |
| PfPARE_L83SitedirectedM-R | GACATAATGTCGAGGTAGTAAATAATAATAAGGCAATATAAAAGGACGGAGATAATTATTATATATATAAAAATAGTTGGATTGAGGAG |
| PfPARE_G58CSitedirectedM-F1 | GTAGGTGTTATAATAGCATGTCATTGTATGAATTCTCATGTACGTTTAG |
| PfPARE_G58CSitedirectedM-R1 | CTAAACGTACATGAGAATTCATACAATGACATGCTATTATAACACCTAC |
| PfPARE_G58CSitedirectedM-F2 | CCATCTTTTAATATTGCCTTATTATTATTTACTACTTCTACGTTATGTCTTAAATATTCTAAACGTACATGAGAATTC |
| PfPARE_G58CSitedirectedM-R2 | GAATTCTCATGTACGTTTAGAATATTTAAGACATAACGTAGAAGTAGTAAATAATAATAAGGCAATATTAAAAGATGG |
| PfUba2 Q62LSitedirectedM-F | GCTACAAGAGATTTATACTTTTTTACATCTTTCTTTTTAAATAAAAATAGTCTGTTTAAATTAGTAATATCTATCGTATCTATATCTATAATGTCAATATTCTTA |
| PfUba2_Q62LSitedirectedM-R | TAAGAATATTGACATTATAGATATAGATACGATAGATATTACTAATTTAAACAGACTATTTTTATTTAAAAAGAAAGATGTAAAAAAGTATAAATCTCTTGTAGC |
| PfPARE-ExP-F | AGGAGATATACCATGGATGAAGAGCCAAGGCGG |
| PfPARE-ExP-R | GGTGGTGGTGCTCGAGGACCTGTTCCTCCTGTTTC |
